# Toward an international standardisation roadmap for nanomedicine

**DOI:** 10.1007/s13346-024-01646-2

**Published:** 2024-06-12

**Authors:** Fanny Caputo, Georges Favre, Gerrit Borchard, Luigi Calzolai, Paola Fisicaro, Emeric Frejafon, Nazende Günday-Türeli, Denis Koltsov, Caterina Minelli, Bryant C. Nelson, Jérémie Parot, Adriele Prina-Mello, Shan Zou, François-Xavier Ouf

**Affiliations:** 1 Laboratoire National de métrologie et d’Essais (LNE), ZA Trappes-Elancourt, 29 Rue Roger Hennequin, 78190 Trappes, France; 2 School of Pharmaceutical Sciences, Institute of Pharmaceutical Sciences of Western Switzerland (ISPSO), University of Geneva, Geneva, Switzerland; 3 European Commission, Joint Research Centre (JRC), Ispra, Italy; 4 BRGM.Fr– Service Geological National, Orleans, 45100 France; 5 MyBiotech GmbH, Industriestr. 1b, 66802 Überherrn, Germany; 6 BREC Solutions, 54B Turzyn, Turzyn, 07-221 Poland; 7 National Physical Laboratory, Hampton road, Teddington, TW11 0LW UK; 8 NIST– Material Measurement Laboratory, Gaithersburg, MD USA; 9 SINTEF Industry, Biotechnology and Nanomedicine department, Trondheim, Norway; 10 Laboratory of Biological Characterization of Advanced Materials (LBCAM), Trinity Translational Medicine Institute, School of Medicine, Trinity College Dublin 8, Dublin, Ireland; 11 Metrology Research Centre, National Research Council Canada, Ottawa, ON K1A 0R6 Canada; 12 NanoMesureFrance, LNE, 1 rue Gaston Boissier Paris Cedex 15, Paris, 75724 France

**Keywords:** Nanomedicine, Workshop, Regulatory science, Standardisation, Education, GMP

## Abstract

The French National Metrology Institute (LNE) initiated a series of events to identify priorities for test methods and their harmonisation that directly address regulatory needs in Nanomedicine. One of these workshops entitled *“The International Standardisation Roadmap for Nanomedicine*” held in October 2023 (Paris, France) brought together key experts in the characterisation of nanomedicines and medical products containing nanomaterials, including the Joint Research Centre of the European Commission, SINTEF Industry and the metrology institutes of France, the UK, the USA and Canada, two flagship initiatives of the European Commission (PHOENIX and SAFE-n-MEDTECH Open Innovation Test Beds), representatives of a working party on mRNA vaccines at the European Directorate for the Quality of Medicines (EDQM) and members of international standardisation and pre-normative organisations (including CEN, ISO, ASTM, VAMAS). Two take-home message came out from the discussion. First, developing standard test methods and Reference Materials (RMs) for nanomedicines is a key priority for the European Commission and various stakeholders. Furthermore, there was a unanimous recognition of the need for a unified approach between standardisation committees, regulators and the nanomedicine community. At the USA, Canadian and European level, examples of success stories and of future initiative have been discussed. Future perspectives include the creation of a dedicated Working Group under CEN/TC 352 to consolidate efforts and develop a nanomedicine standardisation roadmap.

**Graphical Abstract:**

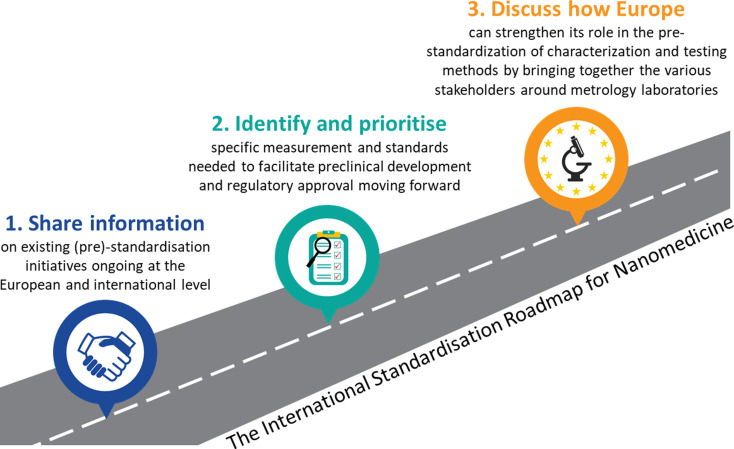

## Objectives of the workshop

Nanomedicines and nano-enabled medical devices therapeutics (defined here as nanotherapeutics) are innovative nanotechnology-based solutions that can impact several health challenges, by delivering therapeutic agents to specific targeted sites in a controlled manner. More than 50 products are now on the market internationally (COVID19 vaccines being the perfect example of a nanomedicine) and > 400 are in clinical trials [1]. This dynamic shows that a significant level of technological maturity has been reached, with increasingly complex nanotherapeutics being developed in research laboratories and considered in clinical trials. In parallel, generic versions of nanomedicine innovator products are starting to be introduced.

Despite the growing number of products in clinical trials and in the clinics, there still is a significant shortage of standard test methods and reference materials that adequately meet the requirements outlined in regulatory guidance documents. The lack of standard test methods and reference materials hampers innovation and complicates the establishment of an appropriate regulatory framework. In the last decade, the international community started working on the development of robust and validated analytical methods, fit-for-purpose reference materials (RMs) and formally recognised consensus standards that will enable streamlining and therefore accelerating the assessment of nanomedicines´quality and safety.

Priorities for methodological developments have been identified in multiple areas of characterization: (i) particle size distribution, including stability and agglomeration, (ii) surface properties, (iii) drug loading and release, (iv) kinetic properties in complex biological media, (v) ADME (absorption, distribution, metabolism, and excretion) parameters, and (vi) interactions with blood and the immune system [2, 3]. The definition of orthogonal approaches, with thorough evaluations of the comparability of results across different techniques and a clear understanding of the measurands involved, would also be needed for the characterisation of such complex critical attributes [4]. In terms of drug product classes, liposomes and lipid based LNPs for mRNA delivery are considered as priorities, followed by metal oxide nanoparticles formulations. In addition to the methodological developments, various actions are needed, including (i) building an ecosystem with networking actions to foster a connection between industrial stakeholders, regulators and characterisation experts; (ii) facilitate the access to available SOPs and standards, and (iii) share information, best practices and perform training to increase the number of GMP accredited laboratories, with a proven expertise in nanomedicine.

In this context, the LNE organised a one-day workshop in Paris on the 13th of November 2023 to discuss the ‘The International Standardisation Roadmap for Nanomedicine’. The objectives of the day were to (i) share information on existing (pre)-standardisation initiatives ongoing at the European and international level (OITB, Euramet, VAMAS, EDQM, ASTM E56, ISO/TC 229, CEN/TC 352), (ii) to identify and prioritise specific measurement and standards needed to facilitate preclinical development and regulatory approval moving forward, and (iii) to discuss how European metrology initiatives could play a more active role bringing together stakeholders involved in the nanomedicine landscape. This perspective provides a summary of the key subjects discussed during the sessions and of the main recommendations provided by the participants. The outcomes will contribute to steer ongoing initiatives and create new ones, as described in the conclusions.

## Open innovation test beds to support the clinical translation of nanotechnology-based health products

An Open Innovation Test Bed (OITB) is a set of entities, providing common access to physical facilities, capabilities and services required for the development, testing and upscaling of nanotechnology and advanced materials in industrial environments. The objective of the Open Innovation Test Beds is to bring nanotechnologies and advanced materials within the reach of companies and users in order to progress from validation in a laboratory (TRL 4) to prototypes in industrial environments (TRL 7). To support the clinical translation of nanotechnology-enabled health products, the European Commission (EC) has financed multiple Open Innovation test Beds (OITBs) initiatives, including the PHOENIX [5] and SAFE-n-MEDTECH OITBs [6]. Services like health technology assessment, scale-up, prototypes and good manufacturing practice (GMP) production, regulatory guidance and pre-clinical characterisation are provided with the aim of identifying promising innovative technologies and reduce their time to market while ensuring their regulatory compliance. They both provide a single-entry point to their services, which is a convenient modality for SMEs and start-ups, as well as academia. As presented by Dr. Günday-Türeli (MyBiotech GmbH), the goal of these initiatives is “to enable the seamless, timely and cost-friendly transfer of nano-pharmaceuticals from lab bench to clinical trials by providing the necessary advanced, affordable and easily accessible services”. Complementarily, Dr. Prina-Mello (Trinity College Dublin) described how nanomedicines can be translated and advanced into innovative clinical products when engaging with the relevant regulatory notified agencies.

Implementing a “fast-track-to-GMP” strategy, including integration of GMP-like procedures during the R&D stage is one of the most important prerequisites for the translational success of nanomedicines with active pharmaceutical ingredients (API).This process should start from the early introduction of laboratory best practice, sound process and experimental design, methodology and instrumentation data logging, protocols verification by complementary techniques, and quality controls and/or assurance on every step of the nanotechnology-enabled health product assessment.

For all types of nanomedicines, intensive characterisation of the product by using standard test methods, customised to special features of the nano-products, dictates the main requirements of scale-up and production. However, there is a lack of fit-for-purpose well-characterised materials and/or RMs to support method validation and product qualification. This is a widely known issues, especially in rapidly evolving health product R&D space where it is critical to establish, as early as possible, a dialogue with notify bodies or regulatory agencies on the definition of objective evidence and quality acceptable criteria, which provide consistent and industrially acceptable assurance on the product intended use. As this poses a significant barrier to the notified body evaluation for the product efficacy and safety, an early technical discussion at the design process phase of the nanotechnology-enabled health product is encouraged, as part of the design verification and validation process of the test methods to adopt for dossier preparation. The setup of quality controls within the academic R&D process is representing a major aspect, which require training and guidance. One of the objectives of the Safe-N-Medtech OITB project was to assist the development and validation of standard operating procedures (SOPs) supporting the industry to overcome the lack of metrological standards. Several inter-laboratory studies (ILS) were carried out (presently under publication) with the precise focus on testing the implementation of new SOPs for the characterisation of nano-enabled products such as nanomedicines in industrial setting with a view to assist and streamline the regulatory assessment process.

## Lipid-based nanoparticles, including RNA-LNP based vaccines: a priority for standardisation in Europe and worldwide

Dr. Calzolai from the Joint Research Centre of the European Commission explained that the development of documentary standards is of strategic importance to strengthen EU technological leadership and to protect EU values [7]. In this context, nanotechnology-based COVID-19 vaccines, as lipid nanoparticles (LNP)-mRNA technologies, have been identified as a key strategic area in the EU standardisation strategy. Another priority for standardisation are liposomal-based medicinal products, which are currently the most numerous nanomedicine formulations representing roughly 50% of the products on the market and in clinical trials [1]. For both LNPs and liposomal formulations, the development of robust analytical methods, their harmonisation and standardisation are needed to accurately measure their properties (quality attributes) and increase the confidence in the produced data.

### mRNA vaccines for human use (MRNAVAC) working party (WP) at the European Pharmacopoeia

In order to assure the quality of mRNA vaccines for human use, and responding to the priorities of the EC, the European Pharmacopoeia of the European Directorate for the Quality of Medicines and Healthcare (EDQM), established an mRNA Vaccines Working Party (November 2022) with Prof. Borchard (University of Geneva) as Chair. This working party is leading a global effort in devising quality standards, with the participation of national control laboratories, mRNA vaccine manufacturers, lipid suppliers, nanofluidics companies, academia, and international regulators. In less than 1 year the group has drafted three general chapters, addressing the mRNA-LNP drug product [8], mRNA drug substance [9] and the DNA template [10]. These texts have been adopted by the European Pharmacopoeia Commission in March 2024, and are available for public consultation from April to August 2024 upon publication in Pharmeuropa [11]. The WP is now focusing on the description and, if possible, the harmonization of test methods and standards for the characterization of these nanomedicines.

### International standardisation efforts

Dr. Parot (SINTEF Industry) and Dr. Koltsov (chair of the ISO/TC 229-*Nanotechnologies*) guided the audience toward the critical steps for the development of a standard test method. This type of standard is a technical document designed to be used as a rule, guideline, or definition. It is a consensus-built, repeatable way of doing something. Standards are created by bringing together experts from all interested parties such as manufacturers, consumers and regulators of a particular material, product, process, or service. The process starts from the identification of a robust measurement protocol (SOP), developed by an expert laboratory, answering a specific analytical need. As explained by Dr. Koltsov, once the creation of a new standardisation project (New Working Item) has been accepted by the committee members, the associated timeline for terminating its development can be quite long (2–4 years). This time is needed to build a consensus method that is accepted by experts and stakeholders in the field. In fact, to reach consensus and global recognition, a standard test method has to go through multiple consultations between national stakeholders and should be validated *via* a rigorous ILS [12].

Multiple examples of international efforts in the field of nanomedicine are ongoing, as presented during the workshop. The US Food and Drug Administration (FDA) under the ASTM International E56 standardisation committee (Nanotechnologies) recently published three standards focused on the measurement of the lipid composition of liposomal drug products, as presented by Dr. Nelson (NIST) [13–15]. Dr. Nelson also expounded on the specific formation and scope of the ASTM E56.08 subcommittee *Nano-Enabled Medical Products* that is directly responsible for the development of pre-competitive documentary standards for nanomedicine applications. E56.08 was formed in 2017 in response to the FDA call for the development of consensus standards to enhance the efficient development and regulatory review of nanomaterial-containing drug product, medical device and in vitro diagnostic submissions. As reiterated throughout the workshop, there exists a rapidly expanding interest in the preclinical use of synthetic lipid-based nanomaterials (liposomes, LNPs, etc.) as drug and gene delivery vehicles in the formulation of next-generation therapeutics and vaccines. However, there exists a critical shortage of standards, mainly test methods and guides, that do not allow for the efficient characterization of the CQAs of current and emerging nanomedicines. There is also a need for standards that are applicable for property determinations in complex biological media and for the assessment of the performance of medical devices that are constructed from or that utilize nanomaterials. ASTM E56.08 is set to rectify this current shortage of nanomedicine standards by initially concentrating on developing standards for liposomal drug products. The lipid composition standards discussed during Dr. Nelson’s talk that describe the quantification of individual lipids in doxorubicin-HCl liposomal drug formulations using three different orthogonal mass concentration (tandem mass spectrometry, evaporative light scattering, charged aerosol) detectors can be used by the pharmaceutical industry to make science-based decisions regarding the quality of their doxorubicin-HCl liposomal drug formulations. These standard test methods are analytically sensitive and selective and are capable of detecting variability in lipid concentrations and ratios at the ng/g to µg/g levels.

Two additional standardisation projects co-led by SINTEF industry and the LNE are ongoing within ASTM International E56, as presented by Dr. Parot. They are focusing on the use of a hyphenated method, combining a fractionation approach called field flow fractionation [16, 17] combined with multiple online detectors to measure the physical properties of liposomal and LNPs formulations, such as size, stability, and drug loading. ISO TC/229 *Nanotechnologies* is also working on the development of documentary standards for nanomedicine, including a vocabulary standard on liposome terminology and a technical specification to quantify the total, encapsulated and free drug in doxorubicin hydrochloride liposomal formulations (ISO/TS 4958). Furthermore, multiple standards have been published or are under development at ISO/TC 229 *Nanotechnologies*, focusing on in vitro toxicity testing, characterisation of nanotechnology-based formulation and on the assessment of their biotransformation in biological fluids. Beyond ISO/TC 229, other ISO technical committees also develop documentary standards relevant to particle-based nanomedicines, most notably ISO/TC 24/SC4 *Particle Characterisation*, ISO/TC 201 *Surface Chemical Analysis* and ISO/TC 276 *Biotechnology*.

### Development of reference materials

RMs are “materials, sufficiently homogeneous and stable with respect to one or more specified properties, which has been established to be fit for its intended use in a measurement process” [18]. Typically, these RMs are designed and intended for method development, inter-laboratory comparisons, verification of instrument performances, or quality control. Their role is crucial in enabling meaningful comparisons of results obtained by testing different sub-samples, conducted from diverse locations, at different times, and using various methods [19]. These RMs are most of the time developed by National Metrology Institutes (NIST, NRC, BAM, LGC, KRISS, etc.) or the JRC at European level. While some reference materials (certified or not) have been developed in recent years in the nano field, fit-for-purpose reference materials, specifically representing nanomedicine clinical formulations available in the market, emerge as a primary focus for validating methods used by regulators, industry, SMEs, and academic laboratories. Dr. Zou from the NRC presented the Canadian metrology institute’s substantial efforts in recent years dedicated to the development of lipid-based nanoscale RMs, encompassing LNPs and liposomes. The NRC has recently issued certificates of analysis for two RMs, certified for their particle sizes, which are the first two reference materials specifically representing clinical drug products based on nanomedicine formulations [20, 21]. This marks a significant advancement in establishing robust standards for the nanomedicine industry and responds to the most urgent demand from players in the field, as a recent survey carried out by the European project MetrINo was able to highlight [22]. With regard to LNPs the size of the particles appears actually as the measurand to be certified as a priority, ahead of the shape of the particles, the RNA payload, the agglomeration state, the impurities, the surface charge, the lipid composition and finally surface coating. In the case of liposomes the trend seems to be slightly different since the measurands to be considered in order of priority for RMs development are the particles size, the drug payload, the shape of particles, the agglomeration state, the surface charge, the surface coating, the lipid composition and finally the impurities. Finally for Metal Oxide nanoparticles (MONPs), the size of particles appears once again as the priority measurand before respectively the agglomeration state, the surface coating, particles’ shape, surface charge and the chemical composition and impurities.

In addition to the development of relevant RMs to meet the priorities identified, another major issue in this field is the knowledge of available reference materials by the players in the field. Various databases are currently available at international level to gather information on available samples, their main characteristics (targeted property/measurand, characterization technique for which they are being considered, any associated reference value and associated uncertainties….) and how to obtain them. But each database has its own particularities, the COMAR international database [23] initially created by the LNE (France) and administered for many years now by the BAM (Germany) only taking into account CRMs and not being limited to the case of nanomaterials, while the Nano-RefMat database [24], hosted by BAM since its creation in the late 2000s as part of a joint project with ISO/TC 229 Nanotechnologies, only targets RMs for the nano field, regardless of whether they are Reference Materials or Certified Reference Materials. Unfortunately this database of great interest has not been updated since 2017 and the BAM is currently looking for a solution to maintain it. The NanoMesureFrance Association, based on this observation, has brought together several of these databases on its website [25] to aggregate the information available on the subject in order to give interested users a means of simplified access to the information available to identify the materials of reference available for their activities.

## SOPs and documentary standards

**Fig. 1:**
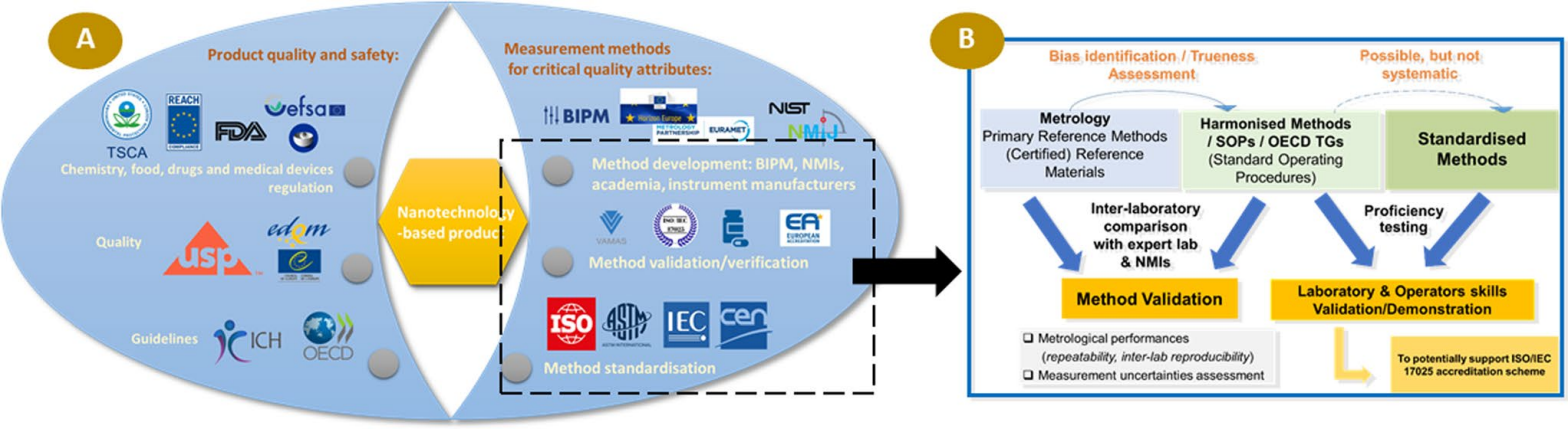
**A**- Fragmentation of the landscape of players involved in the development of validated and harmonized test methods in conjunction with standardization and **B**- Various stages involved in obtaining validated and reliable test methods, and the laboratories competent to deploy them

Beyond the role of RMs in the process of getting trustworthy data, the development of SOPs and documentary standards is key. The work undertaken with liposomal formulations could be duplicated for emerging products including LNP-based vaccines, metal oxide NPs, EVs and nanocrystals. Unfortunately, the standardisation environment (CEN, ASTM, ISO, EDQM, VAMAS) remains very fragmented, and thus there is a need for coordinated and harmonised actions between the different standardisation committees and the metrology communities (Fig. 1).

The EU MetrINo project can serve as an example on the subject to get analytical experts, national metrology institutes, industry, health sector stakeholders and standardization bodies to work together in close collaboration with regulatory stakeholders as a continuation of the reflections initiated within the NanoMesureFrance Association.

Having SOPs and documentary standards available is not sufficient, since they have to be implemented by laboratories to enable the production of trustworthy testing data. It is also crucial to work to make these documents visible in order for stakeholders to be aware of good practices and reference documents in the field, which is very often not the case as numerous surveys on these issues in recent years have shown [3, 26].

It is therefore key that the main standards of interest can be relayed to those responsible for implementing them, whether they are in the research sector or industry. Once again different databases exist on this subject. The NCI-NCL has been working extensively in this direction for many years [27], while ASTM and ANSI have implemented tools to facilitate the identification of existing documents [28, 29], the ANSI Nanotechnology Standards Database being based on a declarative process with all the limitations that may be associated with it. At EU level the MetrINo project has set itself the ambition of ensuring this role in conjunction with the ETPN and the NanoMesureFrance Association by bringing together relevant and useful resources on their respective websites, organising regular events and training sessions for stakeholders (e.g. Workshop Nanomedecine in Paris in June 2022 [30], Webinar: “*Standardisation and Validation Made Simple*” in April 2024 [31]) or to lead regular meetings with the interested community as proposed by the NanoMesureFrance Association through a dedicated Working Group [32].

The requirements for validated protocols and laboratories certified to work under GMP and GLP conditions depends on the pre-clinical phase of the development. Considering the complexity of the multi-structured nanomedicine formulations, it is advisable to proceed by steps of incremental complexity, starting with fairly broad specifications and multiple advance characterisation techniques that may allow an understanding of the correlation between the physical-chemical properties of the products and its biological effects, even if the characterisation methods are not validated and the specifications are quite broaden. Then, at later stages of development specifications will be “tightening” and the requirements of using validated methods to assess the quality attribute of the products become of high relevance. At this step, the selection of service providers for the quality and safety assessment of the nanomedicine formulation is often a challenge for the developers. The choice is usually between CROs or accredited laboratory with a general expertise in pharmaceutical development and quality assessment of pharmaceutical products, that may not have experience with complex nanomedicine formulations and infrastructures with a strong experience with nanomedicine and/or a specific expertise on some specific techniques, that may not, on the other hand be able to guarantee GMP standard (e.g. NCI-NCL, laboratories in the EUNCL consortium, expert academic labs, etc.). In the latter case, it may be possible to qualify the laboratory in the medium/long term by involving both parties (co-development), or considering the possibility of a tech transfer from the expert laboratory to a CRO in charge of routine testing. Accreditation of the testing laboratory according to ISO/IEC 17,025 standards [33] could be another alternative route to provide confidence in the results produced without going as far as the very heavy framework of a GMP certification, the advantage of accreditation being that it requires that the laboratory has demonstrated its skills through the use of RMs and participation in proficiency testing (Fig. 1), unlike the GMP framework. The EU MetrINo project and the NanoMesureFrance Association aim to propose in the future the organisation of inter-laboratory comparisons to offer interested stakeholders a way to provide evidence that they operate competently and generate valid results. This will be an important milestone to support the development of future accreditation scheme in the field of nanomedecine.

The final step is to be able to have these SOPs and standards recognized by the competent authorities, which is the case on the US market with the FDA Recognized Consensus Standards for Medical Devices portal [34], while at European level EMA does not currently offer a similar tool. The participation in the Advisory Board of the MetrINo project of EDQM representative should make it possible to initiate exchanges on these subjects in the coming months, as well as to address the role that the CEN/TC 352 *Nanotechnologies* could have in hosting the set of these discussions by offering a place where the different European actors concerned by the subject could come together.

## How can European metrology support the field of nanomedicine?

Metrology is the science of measurement and its application. It includes all theoretical and practical aspects of measurement, whatever the measurement uncertainty and field of application. It provides reliable and recognised measurements by assuring traceability to common references, the best of which is the International System of Units (SI). Metrology is a key part of the global “quality infrastructure” together with standardization and accreditation.

### Consultative committees on metrology

National metrology institutes worldwide have established the Consultative Committees (CCs) within the BIPM (Bureau International des Poids et Mesures) [35] with the aim to support stakeholders, by providing measurement procedures whose results are traceable to the International System of units and progressing measurement science. As presented by Dr. Fisicaro, the Consultative Committee for Amount of Substance: Metrology in Chemistry and Biology (CCQM) is the closest to nanomedicine applications. The CCQM has recently organised a workshop on particle metrology [36] where among the main outcomes it was recommended to take actions to (i) encourage the development of documentary standards and (ii) create a task group to identify activities the CCQM should undertake with respect to particle metrology over the next ten years, including pilot studies, key comparisons, and cooperative research projects. The task group has been created [37] and over the next two years seeks active engagement with industry and regulators to better understand the important stakeholder needs and gaps in particle metrology that can be addressed by the CCQM. This engagement is critical to inform a credible, useful and pragmatic programme of work.

### Inter-laboratory studies: the VAMAS framework

VAMAS (Versailles Project on Advanced Materials and Standards) ​ [38] aims to promote innovation and the adoption of advanced materials through international collaborations. It provides an international framework to perform pre-normative inter-laboratory comparison exercises, whose technical outcomes often constitute the basis for harmonization of measurement methods and initiation of further standardisation. Related to the field of nanotechnologies and nanomedicine, the Technical Working Area (TWA) 40 on synthetic biomaterials, has recently promoted projects focusing on virus-like particles, including ILS (i) on the measurement of size and number-based size distribution of synthetic virus-like particles, (ii) on the physico-chemical profiling of virus-like particles as RMs for vaccine development and virus particle diagnosis, (iii) on the measurement of intracellular distribution number of virus-like particles per cell and (iv) on quantification of SI-traceable RMs in cells post-transfection. Virus-like particles are receiving much attention within VAMAS for their potentials for nucleic acid delivery in applications including nanomedicines, gene delivery and engineering biology. However, some of the methods tested in these studies will be straightforwardly transferred to a broader range of nanomedicines. Importantly, there is an urgent need to deepen the understanding of the bioactivity and fate of these products in biological systems. These and future VAMAS studies are critical to test the performance of imaging and spectroscopy methods for (i) the localisation, identification, quantification of products in complex biological matrices; (ii) the verification and quantification of the delivery of the active load to the targeted cellular domain; (iii) and the quantification of the resulting therapeutic effect, for example resulting in protein expression or cell death. Participation to the studies is voluntary, but open to all interested laboratories. Participating laboratories benefit from gaining insights into novel materials technologies, accessing a global network of experts and learning best practices on measurement procedures. In addition, the studies provide a means for laboratories to benchmark their measurement capability and review and update their quality framework. There is a need and an opportunity for expert laboratories to design and undertake VAMAS studies on further materials relevant to nanomedicines, including liposomes and lipid nanoparticles to continue develop best measurement practice and thus providing confidence in available methods. The knowledge developed within these interlaboratory exercises will inform nanomedicine-specific documentary standards for the measurement of the various quality attributes, including new measurement methods. Furthermore, VAMAS studies can serve the purpose of testing candidate RMs ahead of formal certification, providing a mean to screen them, refine their scope and improve associated practice. This has the effect of de-risking the production of much needed reference materials in the field while at the same time encourage their uptake. For the process to be effective, it is recommended that VAMAS studies are planned alongside BIPM pilot and key comparison studies and the work of NMI through, for example, national and European metrology programmes.

### EURAMET and the METRINO project

EURAMET, the association of European National Metrology Institutes, (i) supports annual calls for proposals to fund pre-standardisation activities through the European Partnership on Metrology (EPM) [39] and (ii) fosters the establishment of European Metrology Networks in competitive and emerging areas. Among the funded projects, METRINO [40] is focusing on developing and validating traceable measurement methods and RMs for the quality assessment of innovative medicinal products and medical devices containing nanomaterials, including synthetic lipid-based particles such as liposomes and LNPs-RNA, and metal oxide nanoparticles tailored for clinical formulations. Within its first actions, the consortium promoted a survey on metrological needs in the field of nanomedicine, answered by 40 experts and a few key elements have emerged. First, the community expressed the needs for the development of a roadmap on nanomedicine (or roadmaps focusing on different technologies, e.g. mRNA vaccines, liposomes, bio-therapeutics, metal oxide nanoparticles), an effort that would require the joint involvement of complementary stakeholders: experts, standardisation committees, regulatory authorities and the governmental bodies and industry. With regards to the development of reference materials, LNPs and liposomes are considered the highest priorities, while in terms of development of SOPs and standard test methods, the priorities are on the measurements of their Critical Quality Attributes (CQAs) and on methodologies for the detection of NPs in cells and tissues. Finally, there is a strong need for education and training action, since 58% of the survey respondents do not know where to look for SOPs and standards and 69% do not know where to discuss priorities/propose methods for standardisation.

### CEN

CEN is the European Committee for Standardization. It hosts a Technical Committee (TC) in charge of nanomaterials and nanotechnologies, the CEN/TC 352 [41], which is organised into three working groups: WG1 *Measurement, characterization and performance evaluation*, WG3 *Health, safety and environmental aspects* and WG4 *Manufactured nano-objects in food additives*. Dr. Frejafon, the CEN/TC 352 Chair, explained that to date, the TC had not hosted any work specifically on nanomedicine, but that this subject had been identified as a strategic focus for the future, potentially addressing needs associated with nano-enabled medical products and medical devices, but also emerging biotechnologies applications. Therefore, it is recommended to consider setting up a dedicated WG “nanomedecine” in CEN/TC 352 made of nanotechnology measurement experts that work with stakeholders at identifying and prioritising critical quality attributes, and for those to develop standards. To ensure an efficient production of harmonised nanomedicine standards on a global scale, it could be relevant to set up a specific liaison with EMA, EDQM, as well as EU innovation networks and centres on both nanotechnologies (e.g., MetriNo) and nanomedicine (e.g., ETPN). During the March 2024 meeting of CEN/TC352, a formal resolution has been taken to initiate a discussion with EDQM on further collaboration and to move forward jointly on this topic.

## Conclusions and future perspectives

The development of documentary standards and RMs for nanomedicines are key priorities for the European Commission, international metrology institutes, industrial stakeholders and regulators. Thus, numerous (pre)-standardisation efforts are undertaken, mostly focusing on lipid based-nano-formulations. The European Commission has recently updated its standardization strategy [42], it has established a High-Level Forum on European Standardisation and publishes annual priorities. Not surprisingly, in 2022 COVID-19 vaccines and medicines production were amongst the standardization priorities [43].

The standardisation framework is very complex and composed by multiple international standardisation bodies (CEN, ISO, ASTM) and, pharmacopoeia organisations with one or multiple groups specialised in the field of nanomedicine. Ongoing projects and existing standards are often not well-known by the scientific community and the potential users. Finally, communication between committees on ongoing initiatives focusing on similar topics is often lacking. Up to now, the coordination of standardisation efforts and their dissemination have partially relied on experts being involved in parallel on multiple committees.

The creation of a European interest group on standardisation focusing on nanomedicine would be highly desirable to bring together European scientists from academia, expert characterisation platforms, industry, and metrologists. A specific Working Group “nanomedicine” under CEN/TC 352 could be created aiming at mapping the ongoing and existing standardisation efforts developed by ISO, ASTM, CEN, and to propose a standardisation nanomedicine roadmap. This action may lead to the initiation of targeted new projects, notably on standard test methods for the measurement of critical attributes of different types of nanomedicines.

Numerous discussions took place during the Q&A sessions and the two round tables on how to foster interactions between standardisation committees focused on the development of measurement standards, and dedicated organisations specifically associated to the quality assessment of pharmaceutical products such as the International Council for Harmonisation of Technical Requirements for Pharmaceuticals for Human Use (ICH), and the EDQM. Participants recommended that National Metrology Institutes (NMIs) should play the role of connecting the different stakeholders. The creation of a European Metrology Network (EMN) dedicated to medicinal products would be advisable and could constitute the official framework for those exchanges. However, the establishment of such networks requires sustained funds and may take time. In the meanwhile, it was suggested that the newly formed CCQM Task Group on Particle Metrology in collaboration with relevant Joint Research Projects such as MetrINo and/or specific associations of stakeholders (e.g., NanoMesureFrance, or the ETPN- Nanomedicine European Technology Platform) could initiate specific networking actions sharing information on specific initiatives among the different stakeholders belonging to their existing networks.

One rather positive example for the cooperation of different stakeholders is the creation and work of the Working Party on mRNA vaccines for human use. Anchored in the infrastructure of the EDQM, the group of experts from different organisations, regulatory agencies, and even competitors accomplished to draft three important texts that are now published for public comment. There are probably different factors to be considered that lead to their success. The importance of mRNA vaccines for public health and the policy priorities. Second, the vested and shared interest of all experts in the topic and willingness to engage in in-depth discussions. Third, the maintenance of a strict schedule of face-to-face as well as online meetings with defined goals and timelines, which were (mostly) kept. And last but not least, the excellent support of EDQM staff, who also did the heavy lifting of converting the discussion outcomes into publishable texts.

This success story also highlights the key role played by regulatory authorities in shaping the agenda for standard settings and standard recognition. In this context it could be useful to have a formal recognition program for documentary standards similar to that existing CDER Program for the Recognition of Voluntary Consensus Standards Related to Pharmaceutical Quality at FDA [44].

Finally, in terms of new fields to consider, in addition to RNA-LNPs therapeutics and vaccines, liposomes, polymer-based delivery systems, inorganic particles and biotherapeutics have been recently identified as other classes of innovative products to be considered in the future.

## Data Availability

Not applicable.

## Abbreviations

API: Active Pharmaceutical Ingredients
BIPM: Bureau International des Poids et Mesures
CC: Consultative Committees
CCQM: Consultative Committee for Amount of Substance: Metrology in Chemistry and Biology
CQAs: Critical Quality Attributes
EDQM: European Directorate for the Quality of Medicines
EMN: European Metrology Network
GMP: Good Manufacturing Practice
ICH: International Council for Harmonisation of Technical Requirements for Pharmaceuticals for Human Use
ILS: Inter-Laboratory Study
NMI: National Metrology Institutes
OITBs: Open Innovation Test Beds
SOPs: Standard Operating Procedures
TWA: Technical Working Area
VAMAS: Versailles Project on Advanced Materials and Standards
